# A comparative evaluation of various invasion assays testing colon carcinoma cell lines

**DOI:** 10.1038/sj.bjc.6690790

**Published:** 1999-11

**Authors:** N J de Both, M Vermey, W N Dinjens, F T Bosman

**Affiliations:** Department of Pathology, Erasmus University Medical Center, Josephine Nefkens Institute, PO Box 1738, Rotterdam DR 3000, The Netherlands; Department of Pathology, University of Lausanne CH-1011, Lausanne, Switzerland

**Keywords:** Matrigel, confluent fibroblast layers, chicken hearts, orthotopic transplantation

## Abstract

Various colon carcinoma cell lines were tested in different invasion assays, i.e. invasion into Matrigel, into confluent fibroblast layers and into chicken heart tissue. Furthermore, invasive capacity and metastatic potential were determined in nude mice. The colon carcinoma cells used were the human cell lines Caco-2, SW-480, SW-620 and HT-29, and the murine lines Colon-26 and -38. None of the human colon carcinoma cells migrated through porous membranes coated with Matrigel; of the murine lines, only Colon-26 did. When incubated in a mixture of Matrigel and culture medium non-invading cells formed spheroid cultures, whereas invading cells showed a stellate outgrowth. Only the heterogeneously shaped (epithelioid and stellate) cells of SW-480 and SW-620 and the spindle-shaped cells of Colon-26 invaded clearly confluent skin and colon fibroblasts as well as chicken heart tissue. However, when transplanted into the caecum of nude and syngeneic mice, all the lines tested were invasive with the exception of Caco-2 cells. We conclude that the outcome of in vitro tests measuring the invasive capacity of neoplastic cells is largely dependent on the test system used. Invasive capacity in vitro is strongly correlated with cells having a spindle cell shape, vimentin expression and E-cadherin down regulation. In contrast, HT-29 and Colon-38 cells having an epithelioid phenotype were clearly invasive and metastatic in vivo, but not in vitro. © 1999 Cancer Research Campaign


British Journal of Cancer (1999) **81**(6), 934–941
© 1999 Cancer Research Campaign
Article no. bjoc.1999.0790

### A comparative evaluation of various invasion assays testing colon carcinoma cell lines

**NJ de Both[1], M Vermey[1], WN Dinjens[1] and FT Bosman[2]**

[1]Department of Pathology, Erasmus University Medical Center, PO Box 1738, Josephine Nefkens Institute, 3000 DR Rotterdam, The Netherlands; [2]Department of Pathology, University of Lausanne CH-1011, Lausanne, Switzerland

**Summary** Various colon carcinoma cell lines were tested in different invasion assays, i.e. invasion into Matrigel, into confluent fibroblast layers and into chicken heart tissue. Furthermore, invasive capacity and metastatic potential were determined in nude mice. The colon carcinoma cells used were the human cell lines Caco-2, SW-480, SW-620 and HT-29, and the murine lines Colon-26 and -38. None of the human colon carcinoma cells migrated through porous membranes coated with Matrigel; of the murine lines, only Colon-26 did. When incubated in a mixture of Matrigel and culture medium non-invading cells formed spheroid cultures, whereas invading cells showed a stellate outgrowth. Only the heterogeneously shaped (epithelioid and stellate) cells of SW-480 and SW-620 and the spindle-shaped cells of Colon-26 invaded clearly confluent skin and colon fibroblasts as well as chicken heart tissue. However, when transplanted into the caecum of nude and syngeneic mice, all the lines tested were invasive with the exception of Caco-2 cells. We conclude that the outcome of in vitro tests measuring the invasive capacity of neoplastic cells is largely dependent on the test system used. Invasive capacity in vitro is strongly correlated with cells having a spindle cell shape, vimentin expression and E-cadherin down regulation. In contrast, HT-29 and Colon-38 cells having an epithelioid phenotype were clearly invasive and metastatic in vivo, but not in vitro. © 1999 Cancer Research Campaign

**Keywords:** Matrigel; confluent fibroblast layers; chicken hearts; orthotopic transplantation

The most important property of malignant cells is invasive growth. This allows them to leave the compartment to which they normally are restricted, gain access to the connective tissue and the vessels and complete the initial phase of the process of metastasis. It has become clear that in establishing invasive behaviour in addition to intrinsic properties of tumour cells, the microenvironment in which the tumour cells reside also plays a decisive role. Invasive properties are in part reflected by cell shape. In general, epithelioid cells appear less invasive than spindle-shaped cells, and in cell systems in which cell shape can be modulated this appeared to correlate well with invasive behaviour (Sommers et al, 1992; Boyer et al, 1996). A striking example of the influence of the microenvironment is the focal induction of proteases in stromal cells, necessary for the breakdown of the extracellular matrix by tumour cells (Sato et al, 1994; Webb et al, 1994). Another example is the site-specific behaviour of xenografted cancer cells. Human colon cancer cells transplanted to the subcutis of nude mice will neither invade nor metastasize. In contrast, invasion is observed after orthotopic transplantation to the caecal wall, and lymphogeneous as well as haematogeneous metastases occur (Bresalier et al, 1987; Morikawa et al, 1988).

To test invasive capacities of metastasis-competent cells a number of assay systems have been developed. They include confrontation with porous membranes coated with components of the basement membrane, confluent layers of cultured fibroblasts of different origin, chicken heart tissue and orthotopic transplantation in nude mice.

*Received 2 February 1999*
*Revised 10 May 1999*
*Accepted 10 May 1999*

*Correspondence to:* NJ de Both

A main problem in these assays is that they differ significantly in environment, which they provide for the cancer cells and which may cause variation in tumour cell behaviour. In the present study we have tested tumour cell invasion in different assays. The results underline the importance of the composition of the microenvironment for tumour cell behaviour and show that cell morphology is an important determinant of invasive behaviour.

### MATERIALS AND METHODS

### Cell lines

The human colon carcinoma cell lines Caco-2, SW-480, SW-620 and HT-29 were all obtained from the American Type Culture Collection (ATCC).

The murine colon carcinoma cell lines Colon-26 and Colon-38 were obtained from the DCT Repository, National Cancer Institute (Frederick, MD, USA). NIH-3T3 cells were used for making conditioned media and as a positive control for vimentin staining. The E-cadherin-negative bladder carcinoma line J-82 was used as a positive control for invasiveness into Matrigel and was a gift from Dr J Schalken (Department of Urology, University Hospital, Nijmegen, The Netherlands). Human skin fibroblasts (379-P) were obtained from Dr A de Klein (Department of Genetics, Erasmus University, Rotterdam, The Netherlands). Colon stromal fibroblasts were obtained after culture of surgically removed biopsies taken from the mucosa and submucosa.

All established cell lines were cultured in a humidified atmosphere with 5% carbon dioxide in Dulbecco's modified Eagle's medium (DMEM) to which 10% fetal calf serum (FCS) and antibiotics were added. For the Matrigel invasion assay the cells were cultured on RPMI-1640 medium, which contains less sodium-bicarbonate. Primary cells were cultured on F-10 medium (Gibco).

### In vitro testing

### *Matrigel assay*

The assay was carried out as described by Albini et al (1987). Perforated polyvinylpyrrolidone-free polycarbonate membranes of 13-mm diameter (Nucleopore, CA, USA) were used with 8-µm pore size, which is sufficiently large to allow the passage of single carcinoma cells.

To promote attachment all the membranes were first coated on the lower side in 4-well dishes with 15 µl of a 0.1% solution of fibronectin (Sigma, St Louis, MO, USA) in serum-free medium according to Sieuwerts et al (1997). Subsequently half of the membranes were coated with a layer of Matrigel to measure invasion. An amount of 20 µl containing 40 µg Matrigel was applied per membrane surface obtained by dilution of a stock solution with serum-free medium as recommended (Collaborative Biomedical Products, Becton and Dickinson Labware, Bedford, UK). The concentration used prevents preliminary detachment of the Matrigel from the filters and falls within the range of concentrations used by others (see Discussion). The Matrigel was gelatinized at 37°C for 20 min. The other half of the membranes was left uncoated to measure migration.

The membranes were sealed in a sterilized Boyden chemotaxis chamber (Nucleopore Membrane Products, CA, USA) after filling the lower compartment with 220 µl of serum-free NIH-3T3 conditioned medium as attractant. The upper compartment was filled with 200 µl serum-free RPMI medium containing 0.1% bovine serum albumin (BSA), to which $3 \times 10^4$ cells were added.

After incubation for 40 h at 37°C in a carbon dioxide incubator the upper compartments were emptied and in half of the chambers the cells on the upper side of the membranes, which did not contribute to migration c.q. invasion, were removed with a cotton swab. Subsequently the membranes were fixed in 4% glutaraldehyde and stained with a solution of 1% crystal-violet. The number of cells of both the swabbed and unswabbed filters was counted in five different microscopic fields (magnification 400×). Counting of the cells was preferred over the quantitative MTT assay (3-(4,5-dimethylthiazol-2-yl)-2,5-diphenyltetrazolium bromide), since after swabbing often cells remain on the edges of the filter.

The percentage of cells migrating through the uncoated membranes as well as the percentage of cells which had invaded through the Matrigel was determined as the mean of six experiments per cell line.

Since the variances of the percentages of migration and invasion among the cell lines are very heterogeneous, the percentages were analysed after a natural-logarithmic (ln) transformation, which has a homogenizing effect on the variances, as confirmed by the Levene test. After ln-transformation the percentages were broken down by cell line using a one-way analysis of variance (ANOVA), followed by a Student–Newman–Keuls test, in order to find homogeneous subsets of cell lines at the 5% level.

In an additional experiment the cells were cultured in fourfold for 5–7 days in Matrigel varying in concentration from 0.5 to 2 mg $ml^{-1}$. Before gelatinization the Matrigel was diluted with serum containing culture medium and inoculated with cells. During the culture period invasive growth was determined by the stellate outgrowth of the colonies and could be followed in time. After fixation the cultures were stained with crystal-violet and photographed.

### *Invasion in layers of confluent fibroblasts*

An invasion assay was carried out by co-culturing tumour cells with a confluent layer of fibroblasts and by monitoring the invasion of tumour cells into this layer (Fabra et al, 1992). Both human skin and colon fibroblasts were grown to confluency leaving no free space on the bottom of the 24- and 6-well dishes.

A total of $10^4$ carcinoma cells were seeded in the 24-well dishes (diameters 1.5 cm) and $5 \times 10^4$ cells in the 6-well dishes (diameters 3.5 cm). As a rule, the cells adhere to the fibroblasts and remain viable during culture in F-10 medium + 10% FCS. After 4 days the cultures were fixed in methanol–acetic acid (3:1) and stained with Giemsa. Non-invasive cells remained on the surface of the confluent fibroblast layer, whereas invasive cells invaded, pushed away and ultimately replaced the layer of fibroblasts.

### *Chicken heart invasion assay*

The chicken heart assay was carried out as described by Mareel et al (1979). Cells that did not easily attach to the cultured heart fragments were confronted on soft agar during 48 instead of 24 h. Care was taken to avoid the attachment of too large lumps.

Chicken hearts were co-cultivated with tumour cells for 5–6 days in a suspension culture in Rega medium (Gibco) +10% FCS. Growth differences between the various tumour cells had no influence on invasive behaviour. After fixation the invading carcinoma cells could easily be detected in histological sections stained with haematoxylin and eosin (H & E) by their larger size and their hyperchromatic nuclei. The total number of vital chicken heart fragments was counted as well as the number of fragments with attached tumour cells. The latter were subdivided into those with invasion and those with tumour cells only adhering to their surface. The results were statistical treated with the $\chi^2$ test.

### In vivo testing

Female nude mice, 6–8 weeks old, of the NMRI strain were used for both ectopic (subcutaneous) and orthotopic (caecal) transplantations. Before surgery, the mice were anaesthesized with avertine (0.2 mg $g^{-1}$ body weight). After opening the abdominal wall, about $2 \times 10^6$ cells were injected with a microneedle (internal diameter 0.4 mm) into the caecal wall. After a period of 8–20 weeks the animals became cachexic and were killed. Different organs were fixed and screened histologically for invasive growth and the presence of metastases.

### *Immunocytochemistry*

***E-cadherin and vimentin staining.*** Cells were cultured in DMEM +10% FCS on glass slides precoated with 3′-aminopropyltriethoxysilane (Sigma) to enhance cell attachment. Cultured cells were rinsed with phosphate-buffered saline (PBS), fixed in cold acetone and air-dried. The cells were preincubated with 10% normal goat serum for 15 min, followed by a cold incubation overnight with the anti-E-cadherin monoclonal antibody 5H9 (Eurodiagnostica, Arnhem, The Netherlands; dilution × 50–200 in PBS + 1% BSA). For vimentin staining an anti-vimentin monoclonal antibody (Biogenex, Ramon, CA, USA; dilution × 3200) was used. After washing, the cells were incubated for 30 min with a rabbit anti-mouse immunoglobulin serum (Dako, Denmark; dilution × 50), to which 2% normal human serum and 2% normal goat serum were added. After 30-min incubation with the peroxidase–antiperoxidase (PAP) complex (monoclonal antibody P6-38, Sigma; dilution × 300), the cells were stained with 3,3-diamino-benzidine-tetrahydrochloride (Fluka, Oud-Beijerland, The Netherlands) using 0.005% hydrogen peroxide as substrate.

© 1999 Cancer Research Campaign 

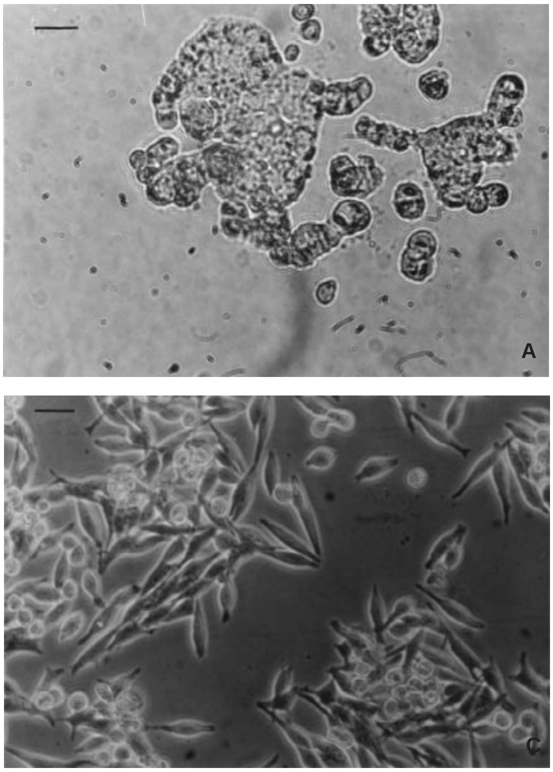

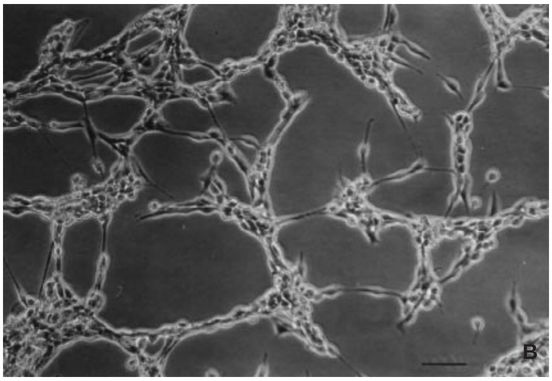

**Figure 1** Modes of growth of colon carcinoma cell lines. (**A**) Compact growth (HT-29); (**B**) Spindle-shaped and dispersed growth (Colon-26); (**C**) Heterogeneous colonies (SW-480). **A** and **C**, 1 bar = 50 µm; **B**, 1 bar = 100 µm. Phase-contrast images

**Table 1** Migration and invasion into Matrigel of various colon carcinoma cell lines

| Cell line | Mean (range) % of invaded cells/total cells | Median | Shape |
|---|---|---|---|
| J-82 (pos. control) | | | |
| Migration | 79.1 (62.9–96.5) | 81.6 | S |
| Invasion | 61.1 (48.9–97) | 52 | |
| Colon-26 | | | S |
| Migration | 75.4 (50–100) | 69.7 | |
| Invasion | 31.2 (11.4–71.7) | 26.7 | |
| Caco-2 | | | E |
| Migration | 4.9 (1.5–10.2) | 4.1 | |
| Invasion | 2.7 (0.8–5.5) | 2.4 | |
| HT-29 | | | E |
| Migration | 6.7 (2.7–12.8) | 5.4 | |
| Invasion | 1.8 (0–2.9) | 2.0 | |
| Colon-38 | | | E |
| Migration | 8.6 (1.6–17.3) | 8.2 | |
| Invasion | 1.8 (0–4.2) | 1.4 | |
| SW-480 | | | E/S |
| Migration | 58.3 (47.3–97.7) | 47.8 | |
| Invasion | 3 (0.9–5.9) | 2.7 | |
| SW-620 | | | E/S |
| Migration | 27.4 (11.4–46) | 25.9 | |
| Invasion | 3.6 (1.1–6.4) | 3.8 | |

A total of 30 000 cells were seeded in the upper compartment of a Boyden chamber on top of membranes, which were either coated with Matrigel or left uncoated. The lower compartment was filled with attractant. After 40-h incubation the percentage was determined of the number of cells present on the lower side of the filter versus the total number of cells of an unswabbed duplo. In six independent assays five microscopic fields (magnification x 400) were counted. For statistical evaluation, see Results. E = epithelioid phenotype; S = spindle-shaped phenotype; E/S = heterogeneously shaped cell population.

For E-cadherin staining of murine colon carcinoma cells, the rat anti-mouse E-cadherin monoclonal antibody (Decma-1, Sigma) was used (dilution × 1500), followed by incubation with a biotinylated rabbit anti-rat immunoglobulin serum (Vector, Brunschwig Chemie, Amsterdam, The Netherlands; dilution × 100). The biotin was visualized for immunofluorescence by avidin–FITC (fluorescein; Vector; dilution × 200).

### RESULTS

Under standard tissue culture conditions the cells showed either an epithelioid (Caco-2, HT-29, Colon-38) or a spindle-shaped morphology (Colon-26), or a mixture of both (SW-480, SW-620). This is illustrated in Figure 1. The epithelioid cells grew in compact colonies in which the cells adhered strongly to each other.

### Matrigel assay

As the results in Table 1 show, the spindle-shaped Colon-26 cells were highly invasive, as were J-82 cells, reaching maximal invasion values of 72 and 97% respectively. These cells also migrated easily through the filters. In contrast, the carcinoma cells with an epithelioid morphology hardly invaded membranes coated with Matrigel, with percentages of invading cells between 0.8 and 5.5 (Caco-2), 0–2.9 (HT-29) and 0–4.2 for Colon-38 cells. The percentage of migrating cells remained lower than 11–13% for Caco-2 and HT-29 cells, and lower than 18% for Colon-38 cells.

Invasion of the lines SW-480 and SW-620 was also low (0.9–6.4%), but their migration through uncoated membranes was much higher, ranging from 47.3% to 97.7% for SW-480 and from 11.4% to 46% for SW-620.

*© 1999 Cancer Research Campaign*

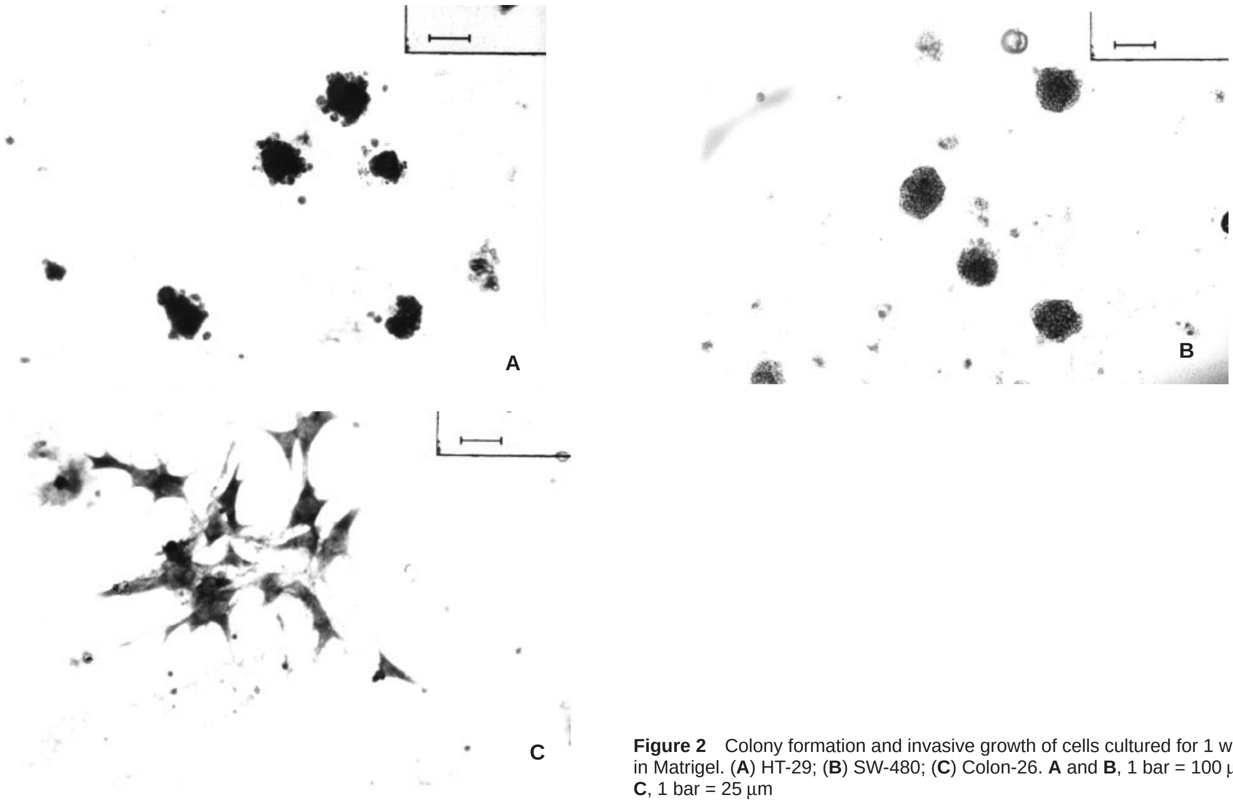

**Figure 2** Colony formation and invasive growth of cells cultured for 1 week in Matrigel. (**A**) HT-29; (**B**) SW-480; (**C**) Colon-26. **A** and **B**, 1 bar = 100 µm; **C**, 1 bar = 25 µm

After a natural-logarithmic (ln) transformation homogeneity of the variances of the percentages of migration and invasion could not be rejected, as tested by the Levene test (respective *P*-values: 0.273 and 0.453).

Using one-way ANOVA analysis significant differences between the cell lines were found. After applying the Student–Newman–Keuls test homogeneous subsets could be distinguished for the different cell lines.

For invasion, three subsets were found, which differed significantly at the 5% level: subset 1, Caco-2, Colon-38, HT-29, SW-480 and SW-620; subset 2, Colon-26; and subset 3, J-82. Between cells belonging to subset 1 there were no significant differences ($P = 0.239$). For migration significantly different subsets were found: subset 4, including Caco-2, Colon-38 and HT-29; subset 5, i.e SW-620; and subset 6, including J-82, Colon-26 and SW-480. Between cells belonging to subsets 4 and 6 no significant differences were found ($P = 0.208$ and 0.446 respectively).

When the cell lines were incubated in the Matrigel diluted with culture medium and varying in concentrations of 0.5–2 mg $ml^{-1}$ the non-invading cells formed well-delineated spheroid colonies (Figure 2 A, B), whereas invading cells showed a stellate outgrowth (Figure 2C).

### Invasion into confluent skin and colon fibroblast layers

The colon carcinoma lines with an epithelioid morphology, i.e. Caco-2, HT-29 and Colon-38 did not invade confluent layers of skin fibroblasts (Table 2, column 1). The cells grew as compact colonies on the surface of the cultured fibroblasts. In contrast, the partly spindle-shaped SW-480 and SW-620 lines and the entirely spindle-shaped Colon-26 cells invaded the fibroblast monolayer. When seeded onto confluent colon fibroblasts the same cell lines proved to be invasive (Figure 3A), whereas the other colon carcinoma cell lines were not invasive in this assay (Table 2, column 2 and Figure 3B).

### Chicken heart assay

Upon confrontation with precultured chicken heart fragments, the invasive properties of cells attached to the fragments could be monitored after 5–6 days in suspension culture. The results are summarized in Table 2, columns 3–5 and Figure 4A–C.

The murine Colon-26 cells were highly invasive in 100% of the attached cases. The partly spindle-shaped SW-480 cells were also invasive (62% of the cases). A similar degree of invasiveness was found for SW-620 cells. Caco-2 and HT-29 cells had invaded a low number of heart fragments (8–10%), notwithstanding the high number of attached proliferating cells. The positive cases only showed superficial invasion. The attached cells mostly grew in lumps (spheroids) with a high number of mitotic figures. The murine colon carcinoma cell line Colon-38 was not invasive either.

The differences between the invasive cell lines SW-480, SW-620 and Colon-26 and the other non-invasive lines were significant in a $\chi^2$ test ($P < 0.005$).

### Heterotopic and orthotopic transplantations (Table 3)

In agreement with earlier observations (de Vries et al, 1995), Caco-2 cells xenografted to nude mice appeared to have a low take rate and grew slowly. From 13 subcutaneous transplantations, nine small tumours were obtained. No caecal tumours were found. The subcutaneous tumours never gave rise to invasive growth or metastases.

Transplanted SW-480 cells formed both subcutaneous tumours and caecal tumours. Some of them showed invasive growth from

© 1999 Cancer Research Campaign

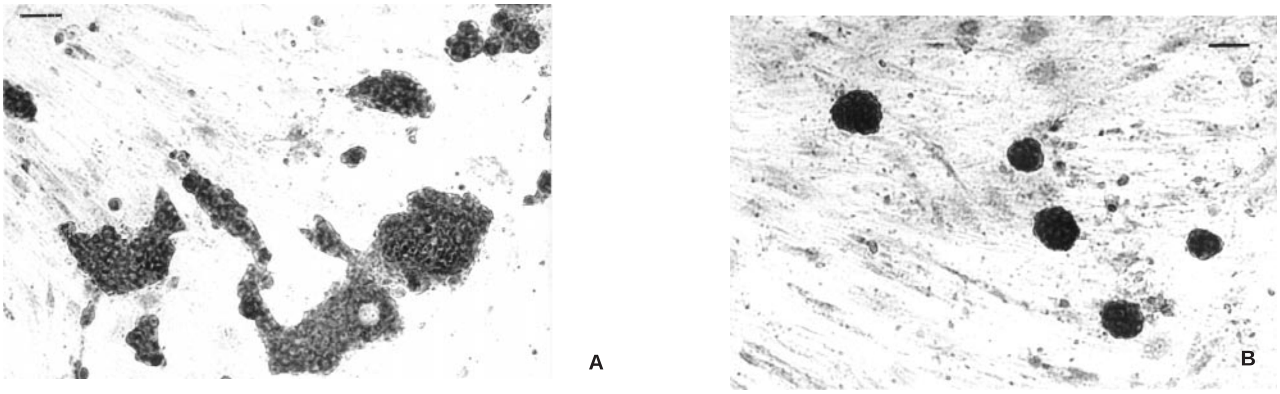

Figure 3 (A) Invasive growth of SW-620 cells in colon fibroblasts. (B) Non-invasive growth of HT-29 cells in colon fibroblasts. Note that the underlying fibroblasts retain their stratified morphology. 1 bar = 100 μm

Table 2 Invasion of colon carcinoma cells in cultured cells

| Cell line | Confluent fibroblasts | | Chicken hearts | | | Shape[a] |
|---|---|---|---|---|---|---|
| | Skin 1 | Colon 2 | Total 3 | Attached 4 | Invasive 5 | |
| Caco-2 | – | – | 82 | 52 | 5 (9%) | E |
| SW-480 | + | + | 29 | 21 | 13 (62%) | E/S |
| SW-620 | + | + | 34 | 26 | 16 (61%) | E/S |
| HT-29 | – | – | 56 | 38 | 3 (8%) | E |
| Colon-26 | + | + | 27 | 15 | 15 (100%) | S |
| Colon-38 | – | – | 18 | 10 | 0 (0%) | E |

Fibroblasts, derived from skin or colon tissue, were cultured to confluent monolayers. A total of 10 000–50 000 colon carcinoma cells were seeded upon these layers and cultured for 4 days. Precultured chicken heart fragments were confronted with small clumps of colon carcinoma cells and cultured for 5–6 days. Invasion of cells could be verified histologically. The invasion of the lines SW-480, SW-620 and Colon-26 differed significantly from the other cell lines (*P*-value < 0.005). [a]See note Table 1.

caecal transplants. The frequency of metastases appeared to be low (one out of six caecal tumours). SW-620 cells showed a lower take rate in subcutaneous and caecal transplantations than SW-480 cells.

For HT-29 cells the take rate was the highest, for both subcutaneous (100%) and for caecal xenografts (58%). From caecal tumours (Figure 5A) the cells invaded the surrounding smooth muscle and lymphatic vessels (Figure 5B); often they formed metastases in the mesenteric lymph nodes. Metastasis to the pancreas, liver, kidneys and lungs was also found (Figure 5C). In 31% of the xenografted animals one or more metastases were detected.

Four subcutaneous and caecal transplantations were carried out with the murine cell lines Colon-26 and Colon-38 in Balb/c and C57/Black mice. Tumours from both cell lines formed metastases, confirming data of Corbett et al (1985). Colon-26 cells were the only cells capable of invading underlying muscle tissue when transplanted subcutaneously.

Staining with anti-E-cadherin antibodies showed that the cells growing in compact colonies with an epithelioid morphology such as Caco-2, HT-29 and Colon-38 were E-cadherin-positive (Figure 6A), whereas SW-480 and SW-620 were negative. Also Colon-26, which has a striking spindle-shaped morphology was negative.

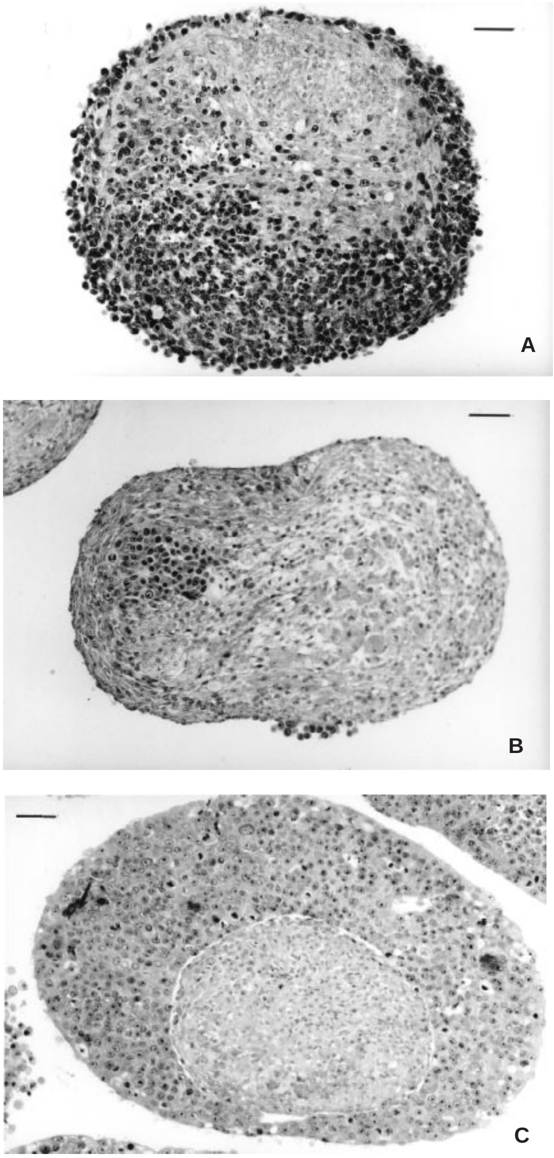

Figure 4 Testing invasive growth in precultured chicken hearts. (A) Highly invasive growth of Colon-26 cells; (B) Invasion of SW-620 cells; (C) Non invasive growth of HT-29 cells, which engulf chicken heart tissue. 1 bar = 50 μm

© 1999 Cancer Research Campaign

**Table 3** In vivo invasion and metastasis

| | Subcutaneous transplantation | | | | Caecal transplantation | | | | |
|---|---|---|---|---|---|---|---|---|---|
| **Cell line** | ***N*** | **Take rate** | **Invasive** | **Metastases** | ***N*** | **Take rate** | **Invasive** | **Metastases[a]** | **Average survival (weeks)** |
| CaCo-2 | 13 | 9 (61%) | 0 | 0 | 13 | 0 | 0 | 0 | >31 |
| SW-480 | 8 | 8 (100%) | 0 | 0 | 19 | 6 (31%) | 4 | 1 | 20 |
| SW-620 | 6 | 4 (67%) | 0 | 0 | 8 | 4 (50%) | 2 | 0 | 20 |
| HT-29 | 19 | 19 (100%) | 0 | 0 | 19 | 11 (58%) | 11 | 6 | 9 |
| Colon-26 | 4 | 4 (100%) | 4 | 0 | 4 | 4 (100%) | 4 | 4 | 4 |
| Colon-38 | 4 | 4 (100%) | 0 | 0 | 4 | 4 (100%) | 4 | 3 | 8 |

*n* = total number of transplantations. [a]Detected in different animals.

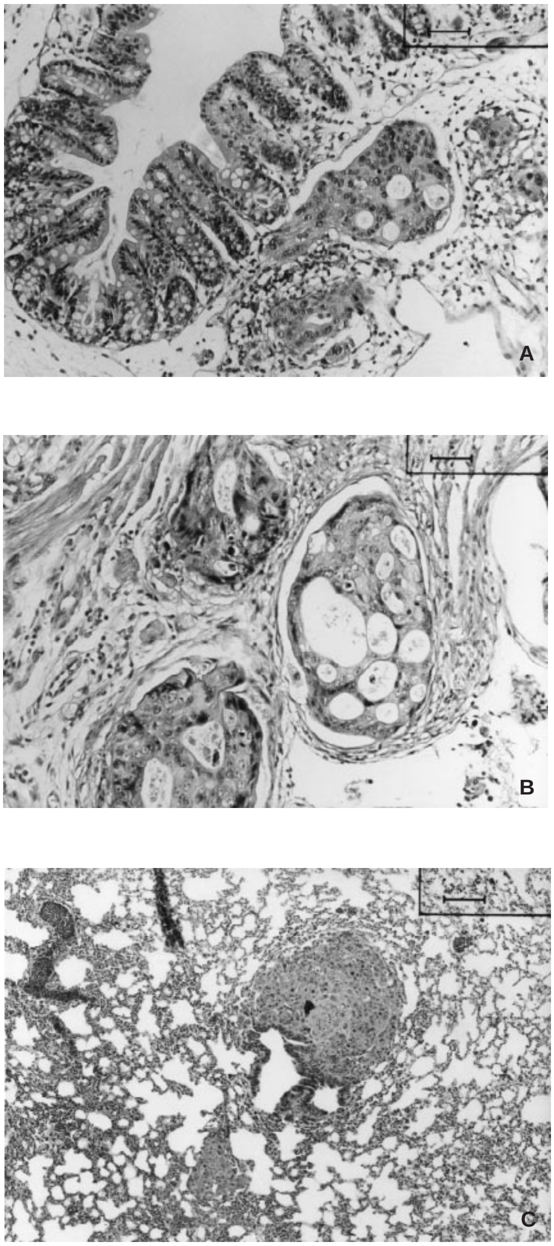

**Figure 5** (**A**) Growth of HT-29 cells in the caecum; (**B**) Invasion of HT-29 cells in lymph vessels of the submucosa; (**C**) Metastasis of HT-29 cells in lung tissue. **A** and **B**, 1 bar = 50 μm, **C**, 1 bar = 100 μm

Anti-vimentin stained not only J-82, Colon-26, SW-480 and SW-620 cells, but to our surprise also HT-29 and Colon-38 cells (Figure 6B). Caco-2 cells were negative. Therefore, a correlation exists between vimentin expression, down-regulation or absence of E-cadherin and invasion in vitro. However, co-expression of E-cadherin and vimentin allows invasion in vivo, but not in vitro.

### DISCUSSION

For colorectal carcinoma cells the step-wise development of malignancy is paralleled by mutations in the APC, K-*ras*, p-53 and DCC genes (Kinzler and Vogelstein 1996). In the majority of cases the lethal course of these tumours is not due to the primary tumour, but to the formation of metastases. In colon carcinoma as yet no genes have been identified, which, as master genes, could be directly responsible for the proteolytic breakdown of the sub-cellular matrix, cell migration, in- and extravasation, homing and secondary outgrowth.

In the study of invasion, in vitro models are often used, since they are fast and convenient. However, they do not allow a complete analysis of the complex in vivo situation, in which host–tumour cell interactions play a role. This led us to compare different in vitro invasion assays with in vivo tumour cell behaviour.

In our experiments the invasiveness of tumour cells in a cell-free matrix did not correlate with in vivo results, confirming results of Noel et al (1991). Since migration is a prerequisite for invasion, the question remains if invasion of epithelioid cells in a Matrigel assay is hampered by low migration rates. Apparently the mode of growth of most colon carcinoma cells showing strong mutual adhesion results in low migration rates, and consequently in low invasion into Matrigel. However, correction of invasion rates for migration rates (Sieuwerts et al, 1997) would lead to hypothetical high invasion rates for most colon carcinoma cells. Also, lowering the concentration of Matrigel could theoretically favour invasion. In the literature the concentration of Matrigel used varies from 20 to 500 μg per filter. In general, low concentrations are used during short running times and higher concentrations during longer running times (Albini et al, 1987). Most authors use a concentration between 25 and 50 μg $ml^{-1}$ (Kath et al, 1991; Sommers et al, 1992; Sunitha et al, 1994), but sometimes much higher concentrations are used as well for colon carcinoma cell lines (Chao et al, 1996). Since the mode of growth of the cells cultured as spheroids in varying concentrations of Matrigel correlated well with the results obtained with the coated filters, it is unlikely that the results of the quantitative measurements are due to too high concentrations of Matrigel.

*© 1999 Cancer Research Campaign*

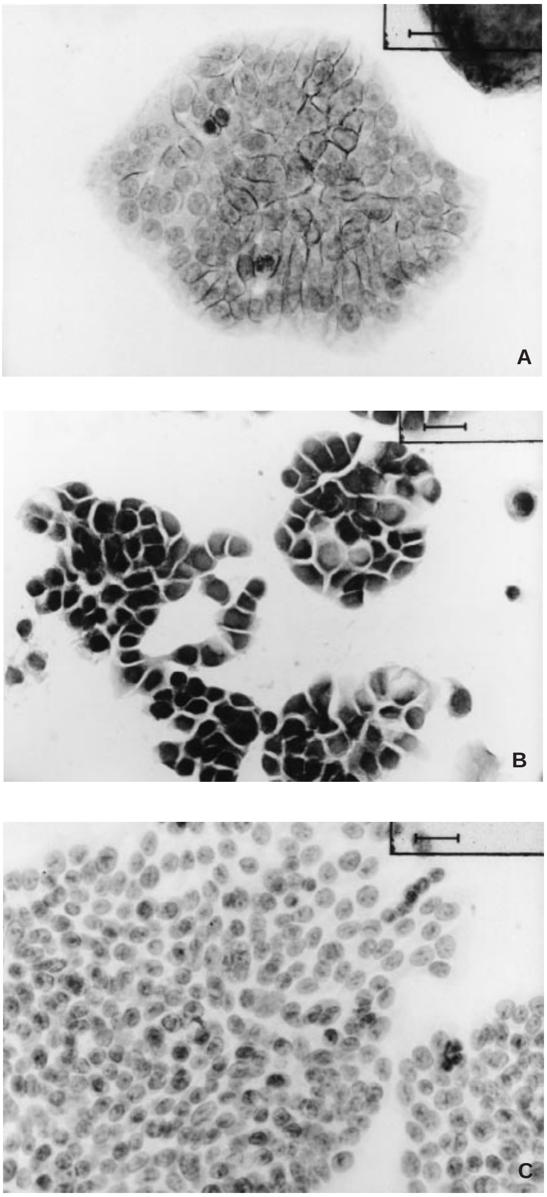

**Figure 6** (**A**) E-cadherin staining in colonies of HT-29 cells; (**B**) Vimentin staining in HT-29 cells; (**C**) Control. 1 bar = 50 μm

Only the partly spindle shaped lines SW-480, SW-620 and Colon-26 invaded skin and colon fibroblasts, but the other carcinoma lines did not. No differences in cancer cell behaviour on skin versus colon fibroblasts were observed, which is in contrast with the postulated organ specificity of stromal elements (Fabra et al, 1992). Moreover, the most aggressive cell line in vivo, HT-29, was not invasive in colon fibroblasts.

The results of the invasion of chicken hearts showed a similar correlation with cell shape. The spindle-shaped cells appeared to be much more invasive in muscle tissue than the epithelioid cells. This was observed both in human and murine colon carcinoma cells. In this connection it is worthwhile to test still more sophisticated three-dimensional and heterologous culture systems, which have recently been developed and which allow study of the interaction of tumour cells with fibroblasts, endothelial cells or immuno-competent cells (Kunz-Schughart et al, 1998).

The most aggressive cells originating from epithelial tumours tend to become more fibroblast-like in appearance. This epithelium to mesenchyme transition (EMT) can be temporarily induced by a number of growth factors, i.e. epidermal growth factor, fibroblast growth factor-1, transforming growth factor-α and HGF/SF or components of the extracellular matrix (Boyer et al, 1996; Jeffers et al, 1966). A permanent cellular transition is characterized by the absence due to mutations or down-regulation of the E-cadherin– catenin complex (Vleminckx et al, 1991; Morton et al, 1993; Vermeulen et al, 1995; Sparks et al, 1998). Restoring the E-cadherin and α-catenin levels by gene or chromosome transfer results in reappearance of the epithelioid phenotype (Ewing et al, 1995, Meiners et al, 1998).

In a number of established carcinoma cell lines the EMT transition leads to a constitutive expression of the mesenchymal marker vimentin (Gilles et al, 1998; Polette et al, 1998), often correlated with invasive growth, lack of desmosomes and absence of steroid receptors (Thompson et al, 1992). In the colon carcinoma cells tested in our experiments a correlation also exists between vimentin expression and invasive growth, but for invasion in vitro the absence of E-cadherin is also required.

In vivo experiments are carried out with nude mice. In the subcutis transplanted tumour cells rarely show invasive growth, due to restrictions of the local stromal environment. In contrast, in orthotopic transplantation (in the caecum) the tumour cells are confronted with organ-specific stromal cells (de Vries et al, 1995). Our results show that all colon carcinoma cells that grow as caecal xenografts are also invasive in vivo. The discrepancy between the results obtained in vivo and in vitro may be explained by the transient down-regulation of adhesion molecules in vivo (Mareel et al, 1991). After colonization of other organs these molecules are up-regulated again and the cells can resume cellular differentiation as seen in many metastases. In a previous study it was shown that clonal overgrowth rapidly takes place in transplants of virally tagged colon carcinoma cell lines (de Both et al, 1997). Such a clonal selection may favour the outgrowth of a cell population prone to invasive growth by down-regulation of adhesion molecules.

In conclusion, our data indicate: (1) consistency between the in vitro assays tested as regards invasive behaviour of cultured neoplastic cells; (2) only the most aggressive spindle-shaped cells are invasive in all test systems used; (3) cells, which are unable to invade Matrigel, can invade chicken heart tissue and confluent fibroblast layers and the latter do not show specificity for the type of fibroblasts used; (4) epithelioid cells not invasive in vitro can be invasive and even metastatic in vivo.

### ACKNOWLEDGEMENTS

We kindly thank Prof Dr M Mareel and co-workers, Department of Experimental Cancerology, University Hospital, Ghent, Belgium for introducing the first author (NJ de B) to the technique of the chicken heart invasion assay. Dr R Verbeek of the Department of Virology, EMCR, was helpful in supplying chicken embryos. Dr P Mulder of the Department of Epidemiology/Biostatistics assisted in the statistical treatment of the data. Ing A Sieuwerts (Department of Internal Medicine) is acknowledged for advice in carrying out the Matrigel assays.

© 1999 Cancer Research Campaign

### REFERENCES

Albini A, Iwamoto Y, Kleinman HK, Martin GR, Aaronson SA, Kozlowski JM and McEwan RN (1987) A rapid in vitro assay for quantitating the invasive potential of tumor cells. *Cancer Res* **47**: 3239–3245

de Both NJ, Vermey M, Groen N, Dinjens WN and Bosman FT (1997) Clonal growth of colorectal carcinoma cell lines transplanted to nude mice. *Int J Cancer* **72**: 1137–1141

Boyer B, Valles AM and Thiery JP (1996) Model systems of carcinoma cell dispersion. *Curr Topics Microbiol Immunol* **213/1**: 179–194

Bresalier RS, Raper SE, Hujanen ES and Kim YS (1987) A new model for human colon metastasis. *Int J Cancer* **39**: 625–630

Chao C, Lotz ML, Clarke AC and Mercurio AM (1996) Function of the integrin $\alpha6\beta4$ in the invasive properties of colorectal carcinoma cells. *Cancer Res* **56**: 4811–4819

Corbett TH, Giswold DP, Roberts BJ, Peckham JC and Schnabel FM (1985) Tumor induction relationships in development of transplantable cancer of the colon in mice for chemotherapy assays with a note on the carcinogen structure. *Cancer Res* **35**: 2434–2439

Ewing CM, Ru N, Morton RA, Robinson JC, Wheelock MJ, Johnson KI, Barrett JC and Isaacs WB (1995) Chromosome 5 suppresses tumorigenicity of PC-3 prostate cancer cells: correlation with re-expression of α-catenin and restoration of E-cadherin function. *Cancer Res* **55**: 4813–4817

Fabra A, Nakajima M, Bucana CD and Fidler IJ (1992) Modulation of the invasive phenotype of human colon carcinoma cells by organ specific fibroblasts of nude mice. *Differentiation* **52**: 101–110

Gilles C, Polette M, Piette J., Delvigne AC, Thompson EU, Foidart JM and Bierembaut P (1998) Vimentin expression in cervical carcinomas: association with invasive and migratory potential. *J Pathol* **180**: 175–180

Jeffers M, Rong S and van de Woude GF (1996) Enhanced tumorigenicity and invasion-metastasis by hepatocyte growth factor/scatter factor-met signalling in human cells concomitant with induction of the urokinase proteolysis network. *Mol Cell Biol* **16**: 1115–1125

Kath R, Jambrosic JA, Holland L, Rodeck U and Herlyn M (1991) Development of invasive growth factor-independent cell variants from primary human melanomas. *Cancer Res* **51**: 2205–2211

Kinzler KW and Vogelstein B (1996) Lessons from hereditary colorectal cancer. *Cell* **87**: 159–170

Kunz-Schughart LA, Kruetz M and Knuechel R (1998) Multicellular spheroids: a three dimensional in vitro system to study tumour biology. *Int J Exp Pathol* **79**: 1–23

Mareel MM, Kint J and Meyvisch C (1979) Methods of study of the invasion of malignant C3H mouse fibroblasts into embryonic chick hearts in vitro. *Virchows Arch B Cell Pathol* **30**: 95–111

Mareel MM, Behrens J, Birchmeier W, de Bruyne GK, Vleminckx K, Hoogewijs A, Fiers WC and van Roy FM (1991) Down regulation of E-cadherin expression in Madin-Darby canine kidney (MDCK) cells inside tumors of nude mice. *Int J Cancer* **47**: 922–928

Meinders S, Brinkmann V, Naundorf H and Birchmeier W (1998) Role of morphogenetic factors in metastasis of mammary carcinoma cells. *Oncogene* **16**: 9–20

Morikawa K, Walker SM, Nakajima M, Pathak S, Milburn Jessup J and Fidler IJ (1988) The influence of organ microenvironment on growth selection and metastasis of human colon cancer cells in nude mice. *Cancer Res* **48**: 6863–6871

Morton RA, Ewing CM, Nagafushi A, Tsukita S and Isaacs WB (1993) Reduction of E-cadherin levels and deletion of the α-catenin gene in human prostate cancer cells. *Cancer Res* **53**: 3585–3590

Noel AC, Calle A, Emonard HP, Nusgens BV, Simar L, Foidart J, Lapiere CM and Foidart JM (1991) Invasion of reconstituted basement membrane matrix is not correlated to the malignant metastatic cell phenotype. *Cancer Res* **51**: 405–414

Polette M, Gilles C, de Bentzmann S, Gruenert D, Tournier JM and Bierembaut P (1998) Association of fibroblastoid features with the invasive phenotype in human bronchial cancer cell lines. *Clin Exp Metastasis* **16**: 105–112

Sato H, Takino T, Okada Y, Cao J, Shinagawa A, Yamamoto E and Seiki M (1994) A matrix metalloproteinase expressed on the surface of invasive tumour cells. *Nature* **370**: 61–65.

Sieuwerts AM, Klijn JGM and Foekens J (1997) Assessment of the invasive potential of human gynecological tumor cell lines with the in vitro Boyden chamber assay: influences of the ability of cells to migrate through the filter membrane. *Clin and Exp Metastasis* **15**: 53–62

Sommers C, Heckford SE, Skerker JM, Worland P, Torri JA, Thompson EW, Byers SW and Gelmann EP (1992) Loss of epithelial markers and acquisition of vimentin expression in adriamycin- and vinblastin-resistant human breast cancer cell lines. *Cancer Res* **52**: 5190–5197

Sparks AB, Morin PJ, Vogelstein B and Kinzler KW (1998) Mutational analysis of the APC/β-Catenin/Tcf pathway in colorectal cancer. *Cancer Res* **58**: 1130–1134

Sunitha I, Meighen DL, Hartman DP, Thompson EW, Byers SW and Avigan MI (1994) Hepatocyte growth factor stimulates invasion across reconstituted basement membranes by a new human small intestinal cell line. *Clin Exp Metastasis* **12**: 143–154

Thompson EW, Paik S, Brunner N, Sommers CL, Zugmaier G, Clarke R, Shima TB, Torri J, Donahue S, Lippman ME, Martin GR and Dickson RB (1992) Association of increased basement membrane invasiveness with absence of estrogen receptor and expression of vimentin in human breast cancer cell lines. *J Cell Physiol* **150**: 534–544.

Vermeulen SJ, Bruyneel EA, Bracke ME, De Bruyne GK, Vennekens KM, Vleminckx KL, Berx GJ, van Roy FM and Mareel MM (1995) Transition of the noninvasive to the invasive phenotype and loss of α-catenin in human colon cancer cells. *Cancer Res* **55**: 4722–4728

Vleminckx K, Vakaet L, Mareel MM, Fiers W and van Roy F (1991) Genetic manipulation of E-cadherin expression in epithelial tumor cells reveals an invasive suppressor role. *Cell* **66**: 107–119

de Vries JE, Dinjens WNM, de Bruyne GK, Verspaget HW, van der Linden EPM, de Bruine AP, Mareel MM, Bosman FT and ten Kate J (1995) In vivo and in vitro invasion in relation to phenotypic characteristics of human colorectal carcinoma cells. *Br J Cancer* **71**: 271–277

Webb G, Baker MS and Nicholl J (1994) Chromosomal localisation of the human urokinase plasminogen activator receptor and plasminogen activator inhibitor type 2 genes: implications in colorectal cancer. *Gastroenterol Hepatol* **9**: 340–343

© 1999 Cancer Research Campaign

## References

[PDF_00549] Albini A., Iwamoto Y., Kleinman H. K., Martin G. R., Aaronson S. A., Kozlowski J. M., McEwan R. N. (1987). A rapid in vitro assay for quantitating the invasive potential of tumor cells.. Cancer Res.

[PDF_00555] Boyer B., Vallés A. M., Thiery J. P. (1996). Model systems of carcinoma cell dispersion.. Curr Top Microbiol Immunol.

[PDF_00557] Bresalier R. S., Raper S. E., Hujanen E. S., Kim Y. S. (1987). A new animal model for human colon cancer metastasis.. Int J Cancer.

[PDF_00559] Chao C., Lotz M. M., Clarke A. C., Mercurio A. M. (1996). A function for the integrin alpha6beta4 in the invasive properties of colorectal carcinoma cells.. Cancer Res.

[PDF_00562] Corbett T. H., Griswold D. P., Roberts B. J., Peckham J. C., Schabel F. M. (1975). Tumor induction relationships in development of transplantable cancers of the colon in mice for chemotherapy assays, with a note on carcinogen structure.. Cancer Res.

[PDF_00552] De Both N. J., Vermey M., Groen N., Dinjens W. N., Bosman F. T. (1997). Clonal growth of colorectal-carcinoma cell lines transplanted to nude mice.. Int J Cancer.

[PDF_00566] Ewing C. M., Ru N., Morton R. A., Robinson J. C., Wheelock M. J., Johnson K. R., Barrett J. C., Isaacs W. B. (1995). Chromosome 5 suppresses tumorigenicity of PC3 prostate cancer cells: correlation with re-expression of alpha-catenin and restoration of E-cadherin function.. Cancer Res.

[PDF_00570] Fabra A., Nakajima M., Bucana C. D., Fidler I. J. (1992). Modulation of the invasive phenotype of human colon carcinoma cells by organ specific fibroblasts of nude mice.. Differentiation.

[PDF_00576] Jeffers M., Rong S., Vande Woude G. F. (1996). Enhanced tumorigenicity and invasion-metastasis by hepatocyte growth factor/scatter factor-met signalling in human cells concomitant with induction of the urokinase proteolysis network.. Mol Cell Biol.

[PDF_00580] Kath R., Jambrosic J. A., Holland L., Rodeck U., Herlyn M. (1991). Development of invasive and growth factor-independent cell variants from primary human melanomas.. Cancer Res.

[PDF_00583] Kinzler K. W., Vogelstein B. (1996). Lessons from hereditary colorectal cancer.. Cell.

[PDF_00585] Kunz-Schughart L. A., Kreutz M., Knuechel R. (1998). Multicellular spheroids: a three-dimensional in vitro culture system to study tumour biology.. Int J Exp Pathol.

[PDF_00591] Mareel M. M., Behrens J., Birchmeier W., De Bruyne G. K., Vleminckx K., Hoogewijs A., Fiers W. C., Van Roy F. M. (1991). Down-regulation of E-cadherin expression in Madin Darby canine kidney (MDCK) cells inside tumors of nude mice.. Int J Cancer.

[PDF_00588] Mareel M., Kint J., Meyvisch C. (1979). Methods of study of the invasion of malignant C3H-mouse fibroblasts into embryonic chick heart in vitro.. Virchows Arch B Cell Pathol Incl Mol Pathol.

[PDF_00595] Meiners S., Brinkmann V., Naundorf H., Birchmeier W. (1998). Role of morphogenetic factors in metastasis of mammary carcinoma cells.. Oncogene.

[PDF_00598] Morikawa K., Walker S. M., Nakajima M., Pathak S., Jessup J. M., Fidler I. J. (1988). Influence of organ environment on the growth, selection, and metastasis of human colon carcinoma cells in nude mice.. Cancer Res.

[PDF_00602] Morton R. A., Ewing C. M., Nagafuchi A., Tsukita S., Isaacs W. B. (1993). Reduction of E-cadherin levels and deletion of the alpha-catenin gene in human prostate cancer cells.. Cancer Res.

[PDF_00605] Noël A. C., Callé A., Emonard H. P., Nusgens B. V., Simar L., Foidart J., Lapiere C. M., Foidart J. M. (1991). Invasion of reconstituted basement membrane matrix is not correlated to the malignant metastatic cell phenotype.. Cancer Res.

[PDF_00608] Polette M., Gilles C., de Bentzmann S., Gruenert D., Tournier J. M., Birembaut P. (1998). Association of fibroblastoid features with the invasive phenotype in human bronchial cancer cell lines.. Clin Exp Metastasis.

[PDF_00611] Sato H., Takino T., Okada Y., Cao J., Shinagawa A., Yamamoto E., Seiki M. (1994). A matrix metalloproteinase expressed on the surface of invasive tumour cells.. Nature.

[PDF_00614] Sieuwerts A. M., Klijn J. G., Foekens J. A. (1997). Assessment of the invasive potential of human gynecological tumor cell lines with the in vitro Boyden chamber assay: influences of the ability of cells to migrate through the filter membrane.. Clin Exp Metastasis.

[PDF_00618] Sommers C. L., Heckford S. E., Skerker J. M., Worland P., Torri J. A., Thompson E. W., Byers S. W., Gelmann E. P. (1992). Loss of epithelial markers and acquisition of vimentin expression in adriamycin- and vinblastine-resistant human breast cancer cell lines.. Cancer Res.

[PDF_00622] Sparks A. B., Morin P. J., Vogelstein B., Kinzler K. W. (1998). Mutational analysis of the APC/beta-catenin/Tcf pathway in colorectal cancer.. Cancer Res.

[PDF_00625] Sunitha I., Meighen D. L., Hartman D. P., Thompson E. W., Byers S. W., Avigan M. I. (1994). Hepatocyte growth factor stimulates invasion across reconstituted basement membranes by a new human small intestinal cell line.. Clin Exp Metastasis.

[PDF_00629] Thompson E. W., Paik S., Brünner N., Sommers C. L., Zugmaier G., Clarke R., Shima T. B., Torri J., Donahue S., Lippman M. E. (1992). Association of increased basement membrane invasiveness with absence of estrogen receptor and expression of vimentin in human breast cancer cell lines.. J Cell Physiol.

[PDF_00634] Vermeulen S. J., Bruyneel E. A., Bracke M. E., De Bruyne G. K., Vennekens K. M., Vleminckx K. L., Berx G. J., van Roy F. M., Mareel M. M. (1995). Transition from the noninvasive to the invasive phenotype and loss of alpha-catenin in human colon cancer cells.. Cancer Res.

[PDF_00638] Vleminckx K., Vakaet L., Mareel M., Fiers W., van Roy F. (1991). Genetic manipulation of E-cadherin expression by epithelial tumor cells reveals an invasion suppressor role.. Cell.

[PDF_00645] Webb G., Baker M. S., Nicholl J., Wang Y., Woodrow G., Kruithof E., Doe W. F. (1994). Chromosomal localization of the human urokinase plasminogen activator receptor and plasminogen activator inhibitor type-2 genes: implications in colorectal cancer.. J Gastroenterol Hepatol.

[PDF_00641] de Vries J. E., Dinjens W. N., De Bruyne G. K., Verspaget H. W., van der Linden E. P., de Bruïne A. P., Mareel M. M., Bosman F. T., ten Kate J. (1995). In vivo and in vitro invasion in relation to phenotypic characteristics of human colorectal carcinoma cells.. Br J Cancer.

